# Role of MicroRNAs in Bone Pathology during Chikungunya Virus Infection

**DOI:** 10.3390/v12111207

**Published:** 2020-10-23

**Authors:** Enakshi Roy, Siddappa N. Byrareddy, St Patrick Reid

**Affiliations:** 1 Department of Pathology & Microbiology, University of Nebraska Medical Center, Omaha, NE 68198-5900, USA; enakshi.roy@unmc.edu; 2 Department of Pharmacology and Experimental Neuroscience, University of Nebraska Medical Center, Omaha, NE 68198-5900, USA; 3 Department of Genetics, Cell Biology and Anatomy, University of Nebraska Medical Center, Omaha, NE 68198-5900, USA; 4 Department of Biochemistry and Molecular Biology, University of Nebraska Medical Center, Omaha, NE 68198-5900, USA

**Keywords:** bone remodeling, osteoblastogenesis, osteoclastogenesis, microRNAs, chikungunya virus (CHIKV)

## Abstract

Chikungunya virus (CHIKV) is an alphavirus, transmitted by mosquitoes, which causes Chikungunya fever with symptoms of fever, rash, headache, and joint pain. In about 30%–40% of cases, the infection leads to polyarthritis and polyarthralgia. Presently, there are no treatment strategies or vaccine for Chikungunya fever. Moreover, the mechanism of CHIKV induced bone pathology is not fully understood. The modulation of host machinery is known to be essential in establishing viral pathogenesis. MicroRNAs (miRNAs) are small non-coding RNAs that regulate major cellular functions by modulating gene expression. Fascinatingly, recent reports have indicated the role of miRNAs in regulating bone homeostasis and altered expression of miRNAs in bone-related pathological diseases. In this review, we summarize the altered expression of miRNAs during CHIKV pathogenesis and the possible role of miRNAs during bone homeostasis in the context of CHIKV infection. A holistic understanding of the different signaling pathways targeted by miRNAs during bone remodeling and during CHIKV-induced bone pathology may lead to identification of useful biomarkers or therapeutics.

## 1. Background

Chikungunya virus (CHIKV) is a positive-sense, single-stranded RNA virus of the *Togaviridae* family and *Alphavirus* genus [1]. The genome is 11.8 kb in length and has two open reading frames, the 5′ORF which encodes the non-structural proteins nsP1, nsP2, nsP3, and nsP4, and the 3′ORF, which encodes the structural proteins, capsid (C), envelope (E1 and E2), and two peptides (E3 and 6K). The virus was first isolated in 1952 from the Makonde plateau in southern Tanzania [2,3]. Since its initial outbreak in the mid-1900s, there have been numerous outbreaks of CHIKV infection in Africa. In the late 1950s and early 1960s, there were outbreaks of the disease in Thailand which affected about 31% of the population. In India, CHIKV was first detected in 1963 in West Bengal which was followed by several other outbreaks between 1964 and 1973. In 2004, there was a CHIKV outbreak in Kenya. In 2005, the largest CHIKV outbreak occurred in India which resulted in about 1.5 million infections. Another CHIKV outbreak occurred in 2005 in Réunion island which affected approximately one-third of the island’s population. Other cases of large-scale epidemics were in Mauritius in 2006, Gabon in 2007, and Thailand in 2008. In 2010, an autochthonous transmission of CHIKV was recorded in southern France. In 2011, an outbreak occurred in the Republic of Congo. Beginning in 2013, this virus spread to the Americas, in part due to travel from affected regions [4,5,6]. In 2014, outbreaks occurred in Martinique-Guadeloupe and French Polynesia. To date, three CHIKV genotypes have been identified, which are West African, Asian, and the East Central and South Asian (ECSA) lineages [5]. The sub Indian Ocean lineage (IOL) is a descendant of the ECSA lineage. CHIKV is mainly transmitted by *Aedes (Ae.)* species mosquitoes [4]. The word “chikungunya” means “that which bends up” in Makonde language to describe the stooped posture of infected patients due to musculoskeletal pain. CHIKV causes CHIK fever (CHIKF), a disease that is typically accompanied by fever, headache, rash, and arthralgia [7]. Although the mortality rate is low, the morbidity rate is high with 30%–40% of the infected patients developing a chronic stage characterized by debilitating arthritis similar in pathology with rheumatoid arthritis (RA) [7,8]. In rare cases, CHIKV infection also exhibit encephalitic pathology [9,10]. However, the bone related pathologies such as joint pain and arthritis-like conditions are major concerns of CHIKV infection. 

MicroRNAs (miRNAs) are a class of small non-coding single stranded RNAs of 21–25 nucleotides in length which function as post-transcriptional regulators of gene expression [11,12]. miRNAs regulate gene expression by binding to the complementary 3′ untranslated regions (3′ UTR) of target mRNAs and inhibiting their translation either by mRNA degradation or translational repression [13]. Recently, miRNAs have been shown to bind to the 5′ UTR of target genes [14]. The miRNA mediated silencing of the mRNA targets occurs in cytoplasmic processing bodies (P-bodies) [15]. The formation of mature miRNAs is a sequential process. The miRNA gene is transcribed into primary miRNA (pri-miRNA) which is processed into precursor miRNA (pre-miRNA) by a DROSHA/DGCR8 complex [16,17,18]. The pre-miRNA is then exported to the cytoplasm where it undergoes further processing by DICER/TRBP/PACT enzyme complex to generate short double-stranded (ds) RNA [19]. Usually, the RNA strand with lower stability at the 5′ end is integrated into the RNA-induced silencing complex (RISC) and become a mature miRNA, while the strand with higher stability at the 5′ end is degraded [20]. The miRNA-induced silencing complex (miRISC) then binds to the 3′ UTR or 5′ UTR of the target mRNA, thus inhibiting its translation. [14]. By affecting gene expression, miRNAs regulate essential physiological and cellular processes including cell cycle, differentiation, proliferation, apoptosis, and immune response [13]. 

Viruses can alter host miRNA levels and the altered expression of those host miRNAs have been associated with the establishment of viral pathogenesis [21]. Here in this review, we highlight the recent studies that have indicated the association of host miRNA with CHIKV infection.

## 2. Role of miRNAs during CHIKV Infection

Viruses are known to hijack host gene expression and modulate fundamental cellular processes to establish infection and pathogenesis. Many studies have demonstrated the role of miRNAs during viral infections [21,22]. Besides having an imperative role in physiological functions, the aberrant expression of miRNAs is associated with pathogenesis of various diseases including viral infections [21,22,23]. Recently, altered expression of several miRNAs (listed in Table 1) has been observed during CHIKV infection [24,25,26,27,28,29,30,31,32].

Given its significance in the regulation of cellular processes, we focus on the recent reports that elucidate the expression levels of different host miRNAs, related target genes, their functions in cellular processes, and role in CHIKV pathogenesis. 

### 2.1. Antiviral Role of miRNAs

The generation of antiviral miRNAs has been observed in many viral infections [21,22]. Computational analysis showed a number of significantly modulated miRNAs in early CHIKV infection are involved in apoptosis and JAK-STAT signaling pathways [32]. Interestingly, the JAK/STAT pathway is known to be one of the key signaling pathways in the interferon (IFN) response against viral infection [33]. Reverse genetic approaches and functional studies in *Ae. aegypti* mosquitoes revealed that increased resistance to Dengue virus (DENV) and Zika virus (ZIKV) infections is mediated by the JAK/STAT pathway [34]. Moreover, CHIKV non-structural protein 2 (nsP2), has been associated with the JAK/STAT pathway [35]. Thus, evaluating the interaction among viral proteins, miRNAs and their involvement in the JAK-STAT pathway holds potential for exploratory studies in CHIKV pathogenesis. 

miRNA profiling in CHIKV-infected human skin fibroblasts showed differential expression of a number of miRNAs in the early stage of CHIKV infection [26]. The miRNAs were predicted to target immune-related signaling pathways including JAK/STAT, MAPK, WNT, and retinoic acid inducible gene I (RIG-I)-like receptor pathways [26]. Interestingly, both JAK/STAT and MAPK pathways have been associated with CHIKV infection [35,36]. Additionally, the WNT signaling pathway can regulate IFN response in flaviviruses [37]. The expressions of hsa-miR-15 and hsa-miR-16 were altered during CHIKV infection [26]. In normal physiology, a number of cellular processes are regulated by hsa-miR-15 and hsa-miR-16 and altered expression of these miRNAs is observed in many other viral infections and diseases [38,39,40,41,42]. Interestingly, hsa-miR-15 and hsa-miR-16 play important roles in inducing apoptosis by targeting the anti-apoptotic protein BCL2 [43]. Additionally, downregulated expression of hsa-miR-15 was found in arthritic synovial tissue, whereas hsa-miR-16 level was high in sera of RA patient [38,44]. rno-miR-32–5 p is a negative regulator of phosphatase and tensin homolog (PTEN) [45]. Thus, understanding the functional relevance of these miRNAs during CHIKV infection would be helpful for the development of novel drug targets.

Microarray analysis in CHIKV infected HEK293T cells revealed a set of 152 differentially regulated miRNAs [28]. Among these, about 65%–70% of the differentially regulated miRNAs were significantly upregulated and the remaining were downregulated. RT-PCR analysis showed that among the upregulated hsa-miRNAs, hsa-miR-744, hsa-miR-638, and hsa-miR-503 were significantly upregulated. Interestingly, 53% of the observed upregulated miRNAs and 45% of the downregulated miRNA were altered in other viral infections including hepatitis B virus (HBV), hepatitis C virus (HCV), human papillomavirus (HPV), and human immunodeficiency virus (HIV). Further analysis of the miRNA pattern demonstrated that the altered miRNAs were members of different miRNA cluster including hsa-miR-17-92, let-7e/99b, hsa-miR-191/425, hsa-miR-106b/25, hsa-miR-23a/24, and hsa-miR-15b/16 clusters which further indicated that these miRNA clusters are co-regulated in response to CHIKV infection [28]. The pathway analysis predicted TGF-β, WNT pathway, endocytosis ubiquitin mediated proteolysis, proteasome and lysosome associated genes, and the cell cycle pathways as targets of the altered miRNAs [28]. Moreover, qRT-PCR results confirmed that the altered miRNAs induced TGF-β genes (mothers against decapentaplegic homolog 6 (SMAD6), JUN, and ski-like protein (SKIL) genes) and endocytosis pathway genes (C-X-C motif chemokine receptor 4 (CXCR4), heat shock cognate 71 kDa protein (HSPA8), adrenoceptor beta 1 (ADRB1)), but inhibited genes involved in cell cycle pathways (cell division cycle 27 (CDC27) and CDC23). The cells treated with a TGF-β inhibitor, SB-431542, showed increased CHIKV mediated cell death compared to untreated cells, indicating that TGF-β production is involved in regulating CHIKV infection [28]. TGF-β signaling pathway is a key network in regulating important cellular processes including proliferation, differentiation, apoptosis, epithelial-mesenchymal transition, and migration [46]. Studies show that this pathway is modulated during many viral infections [47]. Importantly, TGF-β pathway is a significant factor in age related complications during CHIKV infection [48]. Thus, miRNA mediated regulation of TGF-β pathway may contribute to host response against CHIKV infection. 

### 2.2. Pro-Viral Role of miRNAs

The ability of miRNAs to regulate gene expression makes them particularly useful for viruses. Often viruses employ cellular miRNAs to target specific genes and downregulate their expression to establish infection [21,22]. The expression of hsa-miR-146a was found to be upregulated in CHIKV-infected human synovial fibroblasts where TRAF6 and IRAK1 were predicted as targets (Table 1) [24]. The expression of these targets was restored in cells transfected with hsa-anti-miR-146a. In addition, overexpression of hsa-miR-146a leads to decreased phosphorylation of NF-кB during infection [24]. The conclusions were similar to another finding which demonstrated that increased expression of hsa-miR-146a enhanced DENV replication by targeting the TRAF6-mediated NF-кB pathway [49]. These results suggested a role of hsa-miR-146a-mediated targeting of the NF-кB pathway during CHIKV pathogenesis. 

In human synovial fibroblasts, an miRNA microarray identified a subset of 26 differentially expressed miRNAs (DEMs) during CHIKV infection (Table 1) [25]. Among the DEMS, expression of hsa-miR-4717-3p, hsa-miR-4299, hsa-miR-1264, and hsa-miR-21-5p were significantly upregulated. AKT3 was predicted as a target for hsa-miR-4717-3p (Table 1). The AKT3 protein is a key regulator of the PI3K/AKT/mTOR signaling pathway which influences various cellular processes including metabolism, growth, proliferation, survival, transcription, and protein synthesis [50]. Moreover, dysregulation of the PI3K/AKT/mTOR pathway has been implicated in many diseases [51,52]. Interestingly, this pathway is moderately activated during CHIKV infection [53]. Thus, hsa-miR-4717-3p may mediate suppression of the robust inflammatory response during CHIKV infection, by targeting AKT3 [25]. In response to an infection, the host cellular system elicits a cytokine mediated immune response, but often due to inefficient pathogen clearance, the immune response results in inflammation [54]. The expression of hsa-miR-4299 was upregulated during CHIKV infection and the suppressor of cytokine signaling 7 protein (SOCS7) was predicted as the target (Table 1) [25]. SOCS7 is known to negatively regulate the STAT3 protein which can either induce IL-6-mediated inflammation or IL-10-mediated suppression of inflammation during CHIKV infection [55]. STAT3 can also promote viral replication and persistence [56]. Thus, hsa-miR-4299 could mediate suppression of SOCS7 which may result in increased STAT3 expression contributing to suppressed immune response during CHIKV infection. As earlier mentioned, viruses employ different strategies to exploit cellular pathways for optimizing chances of survival [57,58]. Agrawal et al. showed that expression of hsa-miR-1264 increased during CHIKV infection and TRIM26 was predicted to be its target (Table 1) [25]. TRIM proteins function as E3 ubiquitin ligase playing an important role in antiviral responses through ubiquitination and proteasomal degradation of IRF3 genes during viral infections [59]. Thus, this indicates that CHIKV infection may lead to hsa-miR-1264-mediated suppression of TRIM26 resulting in depleted antiviral response and enhanced viral replication and persistence [25]. Another E3 ubiquitin ligase, PELI1 was targeted by hsa-miR-21-5p whose expression was upregulated during infection (Table 1) [25]. PELI1 can suppress the NF-кB pathway by ubiquitination and degradation of an NF-кB-inducing kinase (NIK) [60]. As the NF-кB pathway plays a critical role in antiviral response, thus, increased expression of miR-21 during CHIKV infection may contribute to suppression of cytokine signaling by modulating the NF-кB pathway. 

A genome-wide miRNA screen using high throughput RNA sequencing in Huh-7.5.1 cells revealed that alphaviruses have a binding site for hsa-miR-124 [27]. A significant increase in CHIKV production was observed on overexpressing hsa-miR-124, whereas inhibiting hsa-miR-124 led to reduced CHIKV infection. In rare cases, CHIKV infection can result in encephalitic symptoms [9,10]. hsa-miR-124 is predominantly found in neurons and act as a key negative regulator of neuroinflammation [61]. An altered expression of hsa-miR-124 has been associated with brain disease [61]. Thus, it would be interesting to evaluate whether hsa-miR-124 is associated with encephalitic pathology during CHIKV infection. In a study by Nakamachi et al., decreased expression of hsa-miR-124 was observed in fibroblast like synoviocytes (FLS) of patients with RA where hsa-miR-124a contributed to the inflammatory processes in RA pathogenesis by targeting the monocyte chemoattractant protein-1 (MCP-1) and cyclin-dependent kinase-2 (CDK-2) [62]. Thus, hsa-miR-124 may have a role in contributing to inflammation observed during CHIKV infection.

### 2.3. Aberrant Expression of miRNAs in Mosquito Cells during CHIKV Infection

To establish infection and increase virus survival in a mosquito vector, viruses modify the transcriptional profile of the vector [63]. In Aag-2 cells, aae-miR-2944b-5p and aae-miR-2b were observed to have binding sites for the 3′ UTR of CHIKV [29]. When mosquitoes were treated with antagomiR-2944b-5p, they showed more susceptibility to CHIKV infection compared to untreated control which suggested the role of antagomiR-2944b-5p in viral replication. The host vacuolar protein sorting-associated protein 13 (VPS-13) was predicted as a target of aae-miR-2944b-5p. In *Ae. aegypti*, VPS-13 functions in maintaining the mitochondrial membrane potential (MtMP) [29]. Interestingly, studies report that host mitochondria are involved in combating the oxidative stress induced during viral infections [64]. Silencing aae-miR-2944b-5p in Aag-2 cells and infecting with CHIKV increased cellular MtMP, which indicated that aae-miR-2944b-5p interacts with VPS-13 to maintain MtMP [29]. In humans, VPS-13 is involved in post Golgi apparatus sorting and trafficking. Thus, studying the effect of hsa-miR-2944b-5p on VPS-13 expression in human cell lines during CHIKV infection can lead to identification of novel drug target. 

Using next generation RNA sequencing, the expressions of a set of eight miRNAs were found to be altered during CHIKV infection [30]. Among them, the expressions of aae-miR-100, aae-miR-283, aae-miR-305-3p, and aae-miR-927 were significantly upregulated and the expressions of aae-miR-1000, aae-miR-2b, aae-miR-2c-3p, and aae-miR-190-5p were downregulated. Target prediction revealed that aae-miR-100, aae-miR-283, and aae-miR-305-3p commonly affected NK cell-mediated cytotoxicity and protein processing in ER pathways. The analysis also revealed that the metabolic pathways such as the TCA cycle, dorso-ventral axis formation, and valine, leucine, and isoleucine degradation pathways were affected by aae-miR-100 and aae-miR-305-3p. aae-amiR-927 and aae-miR-305-3p were predicted to target SNARE interactions in vesicular transport. Among these, aae-miR-305-3p was predicted to target pathways essential for viral entry such as ECM receptor interaction, endocytosis, and SNARE interactions in vesicular transport. The downregulated aae-amiR-1000, aae-miR-2b, and aae-miR-2c targeted the ribosomal pathway. The upregulated miRNAs targeted genes which encodes for protein tyrosine phosphatase SHP2, ERK1/2, and ubiquitin fusion degradation protein, respectively, whereas the downregulated miRNAs targeted the gene that encodes for the 40S ribosomal protein S16. In another study, next-generation sequencing identified the altered expression of 13 miRNAs during CHIKV infection in Aag-2 cells [31]. Target prediction analysis showed aae-miR-2b targets URM and ubiquitin whereas aae-miR-100 targets CDC42 and sumo-ligase. When cells were treated with aae-antagomiR-2b, increased CHIKV replication was observed. The expression of URM was also significantly high in CHIKV infected cells. Furthermore, CHIKV replication was reduced to 50% in URM knock down cells, indicating that aae-miR-2b-mediated regulation of URM plays a significant role in chikungunya replication. 

Usually in mosquito vectors, viruses establish infection in the salivary gland during a blood meal [63]. For establishing a successful infection, viruses often modulate the gene expression of several proteins in the salivary gland [63]. Next generation sequencing showed that aae-miR-bantam, aae-miR-263a, aae-miR-125, and aae-miR-285 were significantly upregulated in CHIKV-infected *Ae. aegypti* saliva [65]. In *Ae. albopictus* saliva, aal-miR-43b, aal-miR-43a, aal-miR-413a, aal-miR-5, and aal-miR-249 were upregulated [65]. In addition, Aag-2 cells and BHK-21 cells showed decreased CHIKV titers when treated with inhibitors against selected miRNAs indicating the role of salivary gland miRNAs in modulating CHIKV replication. Another study predicted a set of miRNAs that commonly targeted the different genotypes of CHIKV where aae-miR-282-5p, aae-miR-34-3p, and aae-miR-11-5p had binding sites for CHIKV [66]. Moreover, aae-miR-11-5p was conserved among the different lineages of CHIKV and was predicted to target the end of subgenomic untranslated RNA region, thus, indicating that the CHIKV structural proteins may regulate the formation of a miRNA-viral RNA (vRNA) complex at the end of subgenomic RNA untranslated regions, thereby preventing the binding of host translational factors on vRNA.

## 3. Possible Role of miRNAs in Bone Homeostasis in the Context of CHIKV Infection

**Figure 1:**
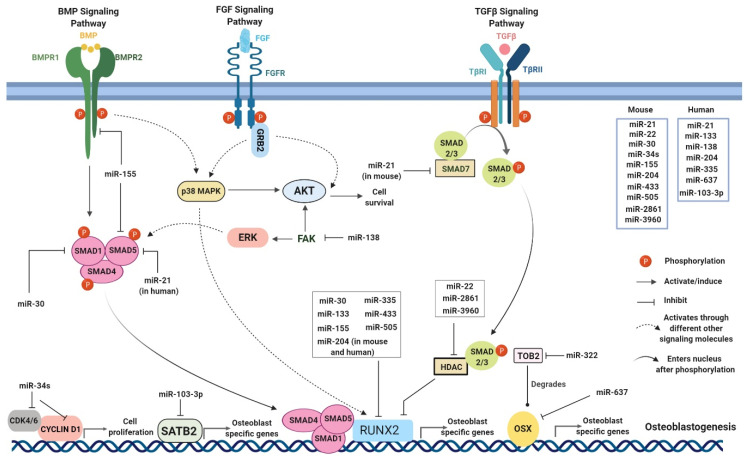
miRNAs regulate different signaling pathways in osteoblastogenesis.

**Figure 2:**
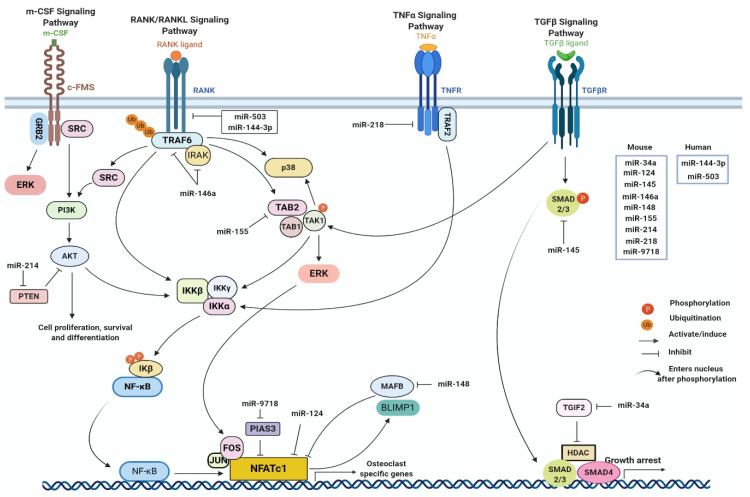
miRNAs regulate different signaling pathways in osteoclastogenesis.

CHIKV infection is associated with bone pathology and it was first indicated by the presence of bony lesions in CHIKV-infected IRF 3/7^-/-^ mice [67]. In CHIKV-infected patients, MRI results showed the presence of erosive arthritis [68]. Bone is one of the most dynamic organs in the body that continuously undergoes remodeling. Bone homeostasis is a highly regulated and complex process involving a fine balance between osteoblastogenesis and osteoclastogenesis [69]. Osteoblastogenesis is the process of bone formation which results from differentiation of mesenchymal stem cells (MSCs) into osteoblastic cell lineage forming the bone cells or osteoblasts (OBs) and later into osteocytes, the mature OBs [70]. Conversely, osteoclastogenesis is the process of bone resorption where the formation of multinucleated osteoclasts (OCs) occurs from the fusion of myeloid precursors which arise by differentiation of hematopoietic stem cells (HSCs) [71]. Many complex processes, signaling pathways, and transcription factors govern osteoblastogenesis and osteoclastogenesis in maintaining normal bone homeostasis (Figure 1 and Figure 2). 

During osteoblastogenesis, the key signaling pathways activated are canonical WNT, NOTCH, Hedgehog, BMP, SMAD, MAPK, and the receptor activator of nuclear factor kβ (RANK), osteoprotegerin (OPG)-and RANK ligand (RANKL) [72,73]. These pathways result in expression of the key transcription factors identified during osteoblastogenesis which are the runt-related transcription factor 2 (RUNX2), and Osterix (OSX) [74,75]. However, there are other transcription factors that also function in bone homeostasis [76,77]. These transcription factors subsequently induce the expression of other osteogenic genes including alkaline phosphatase (ALP), type I collagen (COl-I), osteocalcin (OCN), osteonectin (ON), and bone sialoprotein (BSP) [76,77,78]. Similarly, processes such as OC differentiation from myeloid precursors, maturation, and survival of the OC are regulated by a variety of environmental factors including cytokines, growth factors, and hormones which influence the RANK-RANKL, MAPK, PI3K/AKT, and NF-kβ pathways [72,73]. These signaling pathways in turn regulate the expression of various transcription factors among which nuclear factor of activated T-cells, cytoplasmic 1 (NFATC1) is critical [79,80]. NFATC1 is the major regulator of the early phase of osteoclastogenesis, which induces the expression of other osteoclastic genes in the late phase such as tartrate-resistant acid phosphatase (TRAP), cathepsin k (CTSK), and dendrocyte expressed seven transmembrane proteins (DCSTAMP) [79,81,82,83,84,85]. Conversely, few transcription factors can also negatively regulate osteoclastogenesis [86,87,88,89,90,91].

Bone homeostasis is regulated by an array of transcription factors and complex signaling pathways; thus, understanding how these pathways are modulated during CHIKV infection is of great importance. Recently, a number of studies indicated that miRNAs can regulate both osteoclastogenesis and osteoblastogenesis thereby acting as fine modulators of bone homeostasis [92] (Table 2 and Table 3) (Figure 1 and Figure 2). 

The levels of miRNAs are altered in many bone-related pathological conditions [142]. Furthermore, as mentioned earlier, dysregulated expression of miRNAs has been reported during CHIKV infection (Table 1). Thus, understanding the specific roles of miRNAs in bone homeostasis during CHIKV infection will be critical for the identification of novel biomarkers in altered bone homeostasis during infection and for the development of miRNA-based therapeutics.

In osteogenically differentiated MSCs derived from dental and craniofacial tissues, the expression of hsa-miR-21 was down-regulated, and its overexpression suppressed osteoblastogenesis [94]. SMAD5, the upstream regulator of RUNX2 during osteogenesis, was the target of hsa-miR-21 [94]. However, in mouse osteoblast MC3T3-E1 cells, mmu-miR-21 induced osteogenic differentiation by targeting SMAD7 [95]. Sun et al., also showed that mmu-miR-21 induced osteogenesis as overexpression of mmu-miR-21 resulted in increased mineralization and bone healing properties in a femur fracture model in rats [143]. During CHIKV infection, an upregulated expression of hsa-miR-21-5p was observed, and PELI1, a E3 ubiquitin protein ligase, was predicted as the target [25]. PELI1 has been shown to inhibit the NF-κB signaling pathway, which is an important pathway during osteoclastogenesis [60]. Thus, further studies may be conducted to investigate whether miR-21 can impair osteoclastogenesis during CHIKV infection. FAK signaling pathway acts as a critical signaling pathway in the early stages of osteoblastogenesis [144]. During CHIKV infection, hsa-miR-138-2-3p is upregulated and a number of genes are predicted as targets including MAPK13 [145]. MAPK13 encodes p38 MAPK which plays an important role in bone homeostasis [146]. hsa-miR-138 is also downregulated during osteoblastogenesis as it can target FAK and inhibit the FAK-mediated signaling pathway [145]. Additionally, suppression of hsa-miR-138 expression with antagomiR-138 increased ectopic bone formation in vivo and overexpression of hsa-miR-138 reversed the effects, thus indicating that hsa-miR-138 impairs osteogenic differentiation by targeting FAK and its downstream signaling pathways. However, whether miR-138 regulates any FAK-mediated MAPK-signaling pathway during CHIKV infection is not yet known. Many reports have suggested that joint inflammation is associated with arthritic-like symptoms during CHIKV infection [147]. hsa-miR-146 has been associated with many viral and microbial infections and also with inflammatory conditions such as RA [49,148,149]. An upregulated expression of hsa-miR-146a was observed in synovial fibroblasts during CHIKV infection [24]. Furthermore, TRAF6 and IRAK1 were predicted as targets of hsa-miR-146a [25]. It is known that during viral infections, TRAF6 and IRAK1 activate the NF-кB signaling pathway to produce pro-inflammatory cytokines for combating infection [49]. Additionally, the NF-кB signaling pathway has been shown to play an important role during osteoclastogenesis. However, the effect of hsa-miR-146a on the NF-кB signaling pathway during CHIKV infection remains unknown.

## 4. Conclusions

Understanding the involvement of miRNAs during bone homeostasis in the context of CHIKV infection is of much interest as identification of novel biomarkers and/or development of miRNA-based therapeutics against viral infections is a promising area of research. Several miRNAs already serve as biomarkers and have been associated with pathologies, stages, and/or progression of different diseases. However, the use of miRNAs as biomarkers to diagnose viral diseases is still uncommon. Recently, hsa-miR-181c-5p and hsa-miR-1254 were identified as biomarkers for detection of H1N1 virus influenza [150]. In miRNA-based therapeutics, the developed miRNA either targets the pathogen or host factor during infection. During CHIKV pathogenesis, the two broad areas that can be targeted for drug development are (1) to directly impact virus replication or (2) to modulate host factors to mitigate arthritic-like symptoms caused due to infection. At present, a number of bioinformatic databases and high throughput screens are available to predict miRNA targets during preclinical therapeutic investigations. Additionally, a variety of in vitro cell culture models and in vivo mouse and non-human primate models are available to investigate the efficacy, toxicity, and safety of miRNA therapeutics. A phase 2 clinical trial with miravirsen (locked nucleic acid–modified DNA phosphorothioate antisense oligonucleotide that sequesters the mature hsa-miR-122 in a stable heteroduplex, thereby suppressing its function) in chronic hepatitis C virus (HCV)-infected patients showed reduced HCV RNA levels that persisted beyond the end of active therapy [151]. Another product, RG-101 (an N-acetyl-D-galactosamine-conjugated RNA antagomiR that targets hsa-miR-122 in HCV infected hepatocytes), was used in a clinical trial, which also resulted in undetectable HCV RNA in patients; however, it produced adverse effects due to which the trial was put on hold [152]. Thus, the transition of laboratory findings to clinical applications of miRNA-based diagnostics and therapeutics still remains a challenge and warrants further research. 

## Figures and Tables

**Table 1:** miRNAs altered during Chikungunya virus (CHIKV) infection.

MicroRNAs	Target mRNA	Expression Status (Upregulated/ Downregulated)	References
aae-miR-2b	3′ UTR of ubiquitin related modifier (URM), ubiquitin, and 3′ UTR of CHIKV	Up	[31]
hsa-miR-21-5p	B-cell lymphoma 2 (BCL2), chemokine ligand 1 (CCL1), FASLG, pellino E3 ubiquitin protein ligase 1 (PELI1), interleukin 12 A (IL12A), transforming growth factor beta (TGFβ1)	Up	[25]
hsa-miR-138-2-3p	Tripartite motif containing 26 (TRIM5), TGF-Beta activated kinase 1 (MAP3K7) binding protein 3 (TAB3), tumor necrosis factor receptor superfamily (TNFRSF19), mitogen-activated protein kinase 13 (MAPK13), Apoptotic protease activating factor 1 (APAF1), Forkhead Box O3 (FOXO3)	Up	[25]
hsa-miR-146	Tumor necrosis factor receptor (TNFR)-associated factor 6 (TRAF6), interleukin-1 receptor-associated kinase 1 (IRAK1/2)	Up	[24]
hsa-miR-216a-5p	Cluster of Differentiation 6 (CD6), Janus kinase 2 (JAK2)	Up	[25]
aal-miR-305-3p	ECM receptor interaction, endocytosis, and SNARE interactions in vesicular transport	Up	[30]
hsa-miR-382-3p	Beta-transducin repeat containing E3 ubiquitin protein ligase (BTRC), gap junction protein alpha 1 (GJA1), TRIM8, ubiquitin-conjugating enzyme E2 D2 (UBE2D2)	Up	[25]
hsa-miR-409-3p	DNA topoisomerase 2-beta (TOP2B)	Up	[32]
hsa-miR-491-3p	UBE2B	Up	[25]
hsa-miR-921	nicotinic acid uptake protein (NIAP)	Up	[25]
aal-miR-927	Soluble NSF attachment proteins receptor (SNARE) interactions in vesicular transport	Up	[30]
aae-miR-989	sh2/sh3 adaptor and vacuolar ATP synthase	Down	[31]
hsa-miR-1260a	BCL2 antagonist/killer 1 (BAK1), activating transcription factor 6 beta (ATF6B)	Up	[25]
hsa-miR-1260b	Receptor interacting serine/threonine kinase 1 (RIPK1), E3 ubiquitin-protein ligase NRDP1 (RNF41), suppressor of cytokine signaling 6 (SOCS6), NLR family CARD domain containing 5 (NLRC5), caspase 10 (CASP10)	Up	[25]
hsa-miR-1264	TRIM26, bone morphogenetic protein 2 (BMP2), baculoviral IAP repeat containing 6 (BIRC6), interleukin 6 signal transducer (IL6ST), listerin E3 ubiquitin protein ligase 1 (LTN1), itchy E3 ubiquitin protein ligase (ITCH), MAPK8, SOCS5, UBE2D3	Up	[25]
hsa-miR-3074	Integrin alpha-V (ITGAV), TNF receptor associated factor 3 (TRAF3)	Up	[25]
hsa-miR-4286	Interferon alpha and beta receptor subunit 1 (IFNAR2), interleukin 13 receptor, alpha 1 (IL13RA1), interferon regulatory factor 1 (IRF1), ubiquitin conjugating enzyme E2 Z (UBE2Z), IL6R	Up	[25]
hsa-miR-4299	SOCS7, signal transducer and activator of transcription 5 (STAT5B), TRIM28, mitogen-activated protein kinase kinase kinase 7 (MAP3K7), TAB1, AKT serine/threonine kinase 1 (AKT1), MAP3K11, MAP kinase-activated protein kinase 3 (MAPKAPK3), CAMP responsive element binding protein 1 (CREB1)	Up	[25]
hsa-miR-4443	Interferon regulatory factor 3 (IRF3), mitogen-activated protein kinase kinase kinase 8 (MAP3K8), receptor interacting serine/threonine kinase 3 (RIPK3)	Up	[25]
hsa-miR-4695-3p	CASP8, chemokine C-X-C motif ligand 2 (CXCL2)	Up	[25]
hsa-miR-4717-3p	AKT3, UBE2M, sortilin (SORT1), ring finger protein 213 (RNF213), nerve growth factor receptor-associated protein 1 (NGFRAP1), MAPK10, IL11RA, C-C motif chemokine ligand 4 like 2 (CCL4L2), IFNAR1, IL7R, mitochondrial ubiquitin E3 ligase (MARCH5)	Up	[25]
hsa-miR-4762-5p	Nemo like kinase (NLK)	Up	[25]
hsa-miR-4775	TNFRSF10A, toll-like receptors (TLR1), CASP3, ATF2, TAB2, glycogen synthase kinase 3 beta (GSK3B)	Up	[25]
hsa-miR-4794-5p	UBE2S, STAT1, TRAF5, RIPK1, protein inhibitor of activated STAT 1 (PIAS1), AKT1	Up	[25]
hsa-miR-4878-3p	Diablo IAP-Binding Mitochondrial Protein (DIABLO)	Up	[25]
hsa-miR-5100	UBE2J1, axis inhibition protein 2 (AXIN2), IRAK4	Up	[25]
hsa-miR-5581-3p	MAPK6, IL4R, MAP3K1, CCL18, death inducer-obliterator 1 (DIDO1)	Up	[25]

**Table 2:** The targets and effect of miRNAs on osteoblastogenesis.

miRNA	Target mRNA	Effect on Osteoblastogenesis	References
hsa-miR-10b	SMAD2	Enhances	[93]
hsa-miR-21, mmu-miR-21	SMAD5 (in human), SMAD7 (in mouse)	Inhibits (in human), Enhances (in mouse)	[94,95]
mmu-miR-22	Histone deacetylase 6 (HDAC-6)	Enhances	[96]
mmu-miR-29	ON	Inhibits	[97]
mmu-miR-30	RUNX2, SMAD1, LDL receptor related protein 6 (LRP6)	Inhibits	[98,99]
hsa-miR-34a	Notch1, Notch2, and Jagged-1 (JAG1)	Inhibits	[100]
mmu-miR-34c	Notch1, Notch2, and JAG1	Inhibits	[101]
mmu-miR-34s	CYCLIN D1, CDK4, CDK6, special AT-rich sequence-binding protein 2 (SATB2)	Inhibits	[102]
mmu-miR-103-3p	SATB2	Inhibits	[103]
hsa-miR-133	RUNX2	Inhibits	[104]
hsa-miR-138	Focal adhesion kinase (FAK)	Inhibits	
mmu-miR-145	OSX gene (SP7)	Inhibits	[105]
mmu-miR-155	SMAD5, RUNX2, and bone morphogenetic protein receptor type II (BMPR2)	Inhibits	[106,107]
mmu-miR-183	Heme oxygenase 1 (HMOX-1)	Inhibits	[108]
mmu-miR-204 and hsa-miR-204	RUNX2 (both in mouse and human), ALP (in human), ON (in human)	Inhibits	[109,110]
mmu-miR-221	Zinc finger protein FOG family member 2 (ZFPM2)	Enhances	[111]
mmu-miR-322	Transducer of ERBB2,2 (TOB2)	Enhances	[112]
hsa-miR-335	RUNX2	Inhibits	[113]
mmu-miR-335-5p	Dickkopf WNT signaling pathway inhibitor 1 (DKK1)	Enhances	[114]
hsa-miR-381	WNT5A, frizzled class receptor 3 (FZD3)	Inhibits	[115]
mmu-mir-433	RUNX2	Inhibits	[116]
mmu-miR-495	Aquaporin 1 (AQP1)	Enhances	[117]
mmu-miR-505	RUNX2	Inhibits	[118]
hsa-miR-637	OSX	Inhibits	[119]
mmu-miR-2861	HDAC-5 and homeobox A2 (HOXA2)	Enhances	[120]
mmu-miR-3960	HDAC-5 and HOXA2	Enhances	[120]

**Table 3:** The targets and effect of miRNAs on osteoclastogenesis.

miRNA	Target mRNA	Effect on Osteoclastogenesis	References
mmu-miR-26a	Connective tissue growth factor (CTGF)	Inhibits	[121]
mmu-miR-29 family	G protein-coupled receptor 85 (GPR85), CD93, nuclear factor I A (NFIA)	Enhances	[122]
mmu-miR-29a	RANKL, CXCL12	Inhibits	[123]
mmu-miR-29b	Bcl-2-modifying factor (BMF)	Enhances	[124]
mmu-miR-31	Rhodopsin (RHOA) and the GTPases of the RHO family (Ras-related C3 botulinum toxin substrate 1 (RAC1), RAC2, CDC42, RHOA, and RHOU)	Inhibits	[125]
mmu-miR-34a	TGFB induced factor homeobox 2 (TGIF2)	Inhibits	[126]
mmu-miR-34c	Leucine rich repeat containing G protein-coupled receptor 4 (LGR4)	Enhances	[127]
mmu-miR-124	NFATC1, ras-related protein 27a (RAB27a)	Inhibits	[128,129]
mml-miR-141	EPH receptor A2 (EPHA2)	Inhibits	[130]
hsa-miR-144-3p	RANK, tet methylcytosine dioxygenase 2 (TET2)	Inhibits	[131,132]
mmu-miR-145	SMAD3	Inhibits	[133]
mmu-miR-146a	TRAF6 and IRAK-1	Inhibits	[134]
mmu-miR-148	MAF BZIP transcription factor B (MAFB)	Enhances	[135]
mmu-miR-155	TAB2	Inhibits	[136]
mmu-miR-214	PTEN	Enhances	[137]
mmu-miR-214-3p	TRAF3	Enhances	[138]
mmu-miR-218	TNFR1	Inhibits	[139]
hsa-miR-503	RANK	Inhibits	[140]
mmu-miR-9718	PIAS3	Enhances	[141]
